# Combined SBAS-InSAR and PSO-RF Algorithm for Evaluating the Susceptibility Prediction of Landslide in Complex Mountainous Area: A Case Study of Ludian County, China

**DOI:** 10.3390/s22208041

**Published:** 2022-10-21

**Authors:** Bo Xiao, Junsan Zhao, Dongsheng Li, Zhenfeng Zhao, Dingyi Zhou, Wenfei Xi, Yangyang Li

**Affiliations:** 1 Faculty of Land Resources Engineering, Kunming University of Science and Technology, Kunming 650093, China; 2 Faculty of Road and Construction Engineering, Yunnan Communications Vocational and Technical College, Kunming 650500, China; 3 International Cooperation Department, Kunming Metallurgy College, Kunming 650033, China; 4 Institute of International Rivers and Eco-Security, Yunnan University, Kunming 650500, China; 5 Faculty of Geography, Yunnan Normal University, Kunming 650500, China

**Keywords:** SBAS-InSAR, particle swarm optimization, random forest algorithm, complex mountainous area, landslide, susceptibility prediction

## Abstract

In complex mountainous areas where earthquakes are frequent, landslide hazards pose a significant threat to human life and property due to their high degree of concealment, complex development mechanism, and abrupt nature. In view of the problems of the existing landslide hazard susceptibility evaluation model, such as poor effectiveness and inaccuracy of landslide hazard data and the need for experts to participate in the calculation of a large number of evaluation factor weight classification statistics. In this paper, a combined SBAS-InSAR (Small Baseline Subsets-Interferometric Synthetic Aperture Radar) and PSO-RF (Particle Swarm Optimization-Random Forest) algorithm was proposed to evaluate the susceptibility of landslide hazards in complex mountainous regions characterized by frequent earthquakes, deep river valleys, and large terrain height differences. First, the SBAS-InSAR technique was used to invert the surface deformation rates of the study area and identified potential landslide hazards. Second, the study area was divided into 412,585 grid cells, and the 16 selected environmental factors were analyzed comprehensively to identify the most effective evaluation factors. Last, 2722 landslide (1361 grid cells) and non-landslide (1361 grid cells) grid cells in the study area were randomly divided into a training dataset (70%) and a test dataset (30%). By analyzing real landslide and non-landslide data, the performances of the PSO-RF algorithm and three other machine learning algorithms, BP (back propagation), SVM (support vector machines), and RF (random forest) algorithms were compared. The results showed that 329 potential landslide hazards were updated using the surface deformation rates and existing landslide cataloguing data. Furthermore, the area under the curve (AUC) value and the accuracy (ACC) of the PSO-RF algorithm were 0.9567 and 0.8874, which were higher than those of the BP (0.8823 and 0.8274), SVM (0.8910 and 0.8311), and RF (0.9293 and 0.8531), respectively. In conclusion, the method put forth in this paper can be effectively updated landslide data sources and implemented a susceptibility prediction assessment of landslide disasters in intricate mountainous areas. The findings can serve as a strong reference for the prevention of landslide hazards and decision-making mitigation by government departments.

## 1. Introduction

Landslide refers to the natural phenomenon in which the soil or rock mass on a slope slides down as a mass or as fragments along a certain soft surface or soft zone under the influence of gravity, which is affected by river scour, groundwater activity, rainwater soaking, earthquake, and artificial slope cutting [1]. Approximately 1000 deaths and billions of dollars in property damage are attributed to landslides annually [2]. Under the influence of the Qinghai–Tibet plateau uplift, China’s geological structure and topography are extremely complex and unique, creating favorable geological conditions and abundant material conditions for landslides. In addition, mountainous regions account for approximately 67% of the total land area, and more than 56% of the population resides in mountainous regions [3], making China one of the countries most severely impacted by landslides in the world. For this reason, research into the prediction and evaluation of landslide susceptibility in complex mountainous areas is an effective means of preventing and controlling large-scale landslides. It is possible to develop prevention and control measures by mastering the spatial and temporal landslide distribution and by analyzing the probability of landslide instability given a particular geological environment.

Currently, domestic and international susceptibility evaluation methods for landslide disasters are primarily divided into three categories: empirical models, statistical analysis models, and machine learning models [4]. (1) Empirical model refers to the process by which professional and technical personnel evaluate the occurrence of landslide disasters based on a comprehensive understanding of the research area, informed by expert experience knowledge, and rationale justification. Expert knowledge-based approach [5,6,7] and analytical hierarchy process [8,9,10] are the primary methods. This method is highly subjective and overly dependent on the knowledge and experience of experts. (2) Model for statistical analysis. This technique focuses on analyzing the relationship between slope, aspect, DEM, curvature, lithology, and other environmental factors and landslide hazards. This model creates a landslide prediction model in order to compute the susceptibility index. The information value model [11,12], the logistic regression model [13,14,15], the frequency ratio model [16,17], the weights of evidence method [18,19,20], the certainty factor method [21,22], etc., are widely utilized models. This method has the advantage of strong interpretability, allowing quantitative analysis of the close relationship between landslide and influencing factors and the mutual relationship between these variables. Due to the complexity of landslides and topographic, geomorphic, geological, and other factors, however, it is difficult to adhere to a strictly linear relationship, leading to erroneous evaluation results. (3) Model of machine learning. With the rapid advancement of computer technology, machine learning technology offers a novel approach to studying the susceptibility of landslides. It improves the accuracy of landslide susceptibility evaluation with its potent nonlinear mapping capability. The principal models consist of support vector machine [23,24], random forest [25,26,27], decision tree algorithm [28,29], artificial neural network [30,31,32], etc. The effectiveness of the aforementioned models in predicting and evaluating landslide susceptibility is demonstrated. However, the current evaluation model does not fully account for the nonlinear characteristics of landslide displacement on time series in complex mountainous regions with frequent earthquakes. In reality, the probability of landslide risk varies across time scales. Even if a coupling model is utilized, it is difficult to improve the accuracy of the evaluation. Notably, it is more difficult to precisely control the model accuracy when evaluating the susceptibility to landslide hazards over a large area [33].

Time-series InSAR technology has rapidly advanced in recent years, offering new technical support for the early detection, monitoring, warning, and risk assessment of landslide disasters. In surface deformation monitoring, time-series InSAR technology has a greater monitoring range than other technologies and is not affected by the weather. As such technology is able to obtain images and earth surface data at any time of day or night [34], rendering it a one-of-a-kind remote sensing method for deformation monitoring based on surface data [35]. In complex mountainous regions with frequent landslide disasters, the combination of time-series SBAS-InSAR technology and high-resolution optical remote sensing images can achieve early landslide identification and inverse the slow deformation development process of landslide points in time series [36,37,38]. Random Forest is the most popular and effective algorithm for supervised learning, taking into account the ability to solve regression and classification problems. It has been successfully applied to the evaluation and forecasting of the susceptibility of a variety of geological disasters. In comparison to the conventional optimization algorithm based on a gradient descent, the particle swarm optimization (PSO) algorithm has the advantages of enhanced robustness, excellent scalability, and resistance to local optimum. In comparison to optimization methods based on the natural evolution process, such as evolutionary programming and genetic algorithm, the information sharing mechanism of the particle swarm optimization algorithm accelerates the population convergence to the optimal value [39]. The combination of SBAS-InSAR technology and the PSO-RF algorithm provides a theoretical and practical foundation for the prediction and evaluation of landslide susceptibility.

In light of the issue with existing landslide susceptibility evaluation methods, this paper proposes a combined SBAS-InSAR and PSO-RF algorithm for evaluating the susceptibility of landslide disasters in complex mountainous regions. The specific contents of the research are as follows: (1) Using SBAS-InSAR technology, the deformation variables of existing landslide points and potential landslide points were inversed. According to the deformation variables, high-resolution optical remote sensing images were utilized to locate potential landslide points. (2) Employing traditional geological factors, terrain factors, environmental factors, and human engineering activities, as well as the landslide time series deformation and seismic factors, the PSO-RF algorithm is applied to construct a model, and the landslide susceptibility index is obtained through learning, training, and testing. The algorithm improved the effectiveness and precision of landslide susceptibility evaluation, thereby preventing the loss of life and property caused by inaccurate evaluations.

## 2. Study Area and Dataset

### 2.1. Study Area

**Figure 1:**
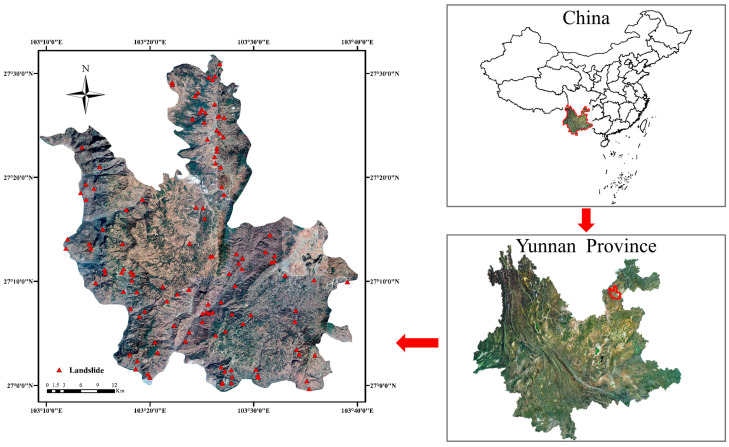
Location of the study area.

Ludian County is located in the northeast of Yunnan Province, southwest of Zhaotong City, on the north bank of Niulanjiang River, spanning between latitude $26^{{}^{\circ}}59^{\prime }\sim 27^{{}^{\circ}}32^{\prime }$ and longitude $103^{{}^{\circ}}09^{\prime }\sim 103^{{}^{\circ}}47^{\prime }$. It shares borders with Zhaotong City in the northeast, Weining County in Guizhou Province in the southeast, and Huize and Qiaojia County on the other side of the Niulan River in the south and west. Complex topography defines this area, with high terrains on either side and a low terrain in the middle, characterized by precipitous mountains and hills, a karst plateau, a mixed mound, a plateau lake basin, and a fault valley dam. The highest point is 3356 m above sea level, and the lowest is 568 m. The elevation of the county seat is 1917 m above sea level. Low-latitude mountain monsoon climate in the study is characterized by distinct dry and wet seasons and pronounced three-dimensional climate characteristics. The annual precipitation average is 923.5 mm. The County area is located on the eastern margin of Lvzhijiang–Xiaojiang north–south tectonic belt of Sichuan-Yunnan meridional tectonic system and the combination site of eastern Yunnan multiple fonts structural system, where tectonic movement is frequent, rock is compressed, deformation is strong. In addition, on 3 August 2014, Ludian County experienced a sudden Mw6.5 earthquake, which induced large-scale landslides and potential geological hazards. Therefore, this paper chooses Ludian County with fragile geological environment, frequent geological disasters, and serious damage as the study area (Figure 1). Figure 1 depicts the locations of landslide cataloging points recorded by the Yunnan Provincial Department of Natural Resources at the triangle-marked landslide points.

### 2.2. Dataset

Sentinel-1A radar satellite data are selected as the update and identification experiment data of the potential landslide points in the study area in order to reduce the decoherence image caused by a long time baseline and to dynamically monitor and update the potential landslide points in the study area. Since Sentinel-1A radar satellite data is currently in orbit. It is available for free and has a brief time baseline. In April 2014, the Sentinel-1A radar satellite was launched. The European Space Agency (ESA) and the European Commission are collaborating on the Sentinel-1A Global Environmental and Safety Monitoring System project (EC). Two C-band synthetic aperture radar-equipped satellites comprise the European Space Agency’s Global Monitoring for Environment and Security (GMES) Earth observation satellite. The satellite orbits at an altitude of 693 km and is equipped with an all-weather radar imaging system. The revisit period for a single star is 12 days, while Sentinel-1A and Sentinel-1B can reduce it to 6 days. The primary modes of operation for Sentinel-1 are Interferometric Wide (IW) Mode and Wave Mode (WM) Mode. There are two extra modes: Strip Map mode (SM) and extra-wide swath mode (EW). On land, the interference wide-band mode is the default mode. In this mode, terrain observation with progression scans SAR (TOPSAR) is used to scan three times back and forth along a strip to obtain three substrips. With a ground resolution of 5 m × 20 m and a width of 250 km, more uniform and high-quality SAR images are generated. The extra-wide swath mode utilizes interference-processing-capable TOPSAR technology similar to the interferometric wide mode. However, the number of strips is increased from three to five, and the resolution is reduced. The resolution on the ground is 20 m × 40 m, and the width is 400 km. The model is primarily utilized in oceans, glaciers, and polar regions where large coverage and short revisit periods are required. This experiment collected 61 ascending orbit images and 58 descending orbit images of Sentinel-1A with the interferometric wide mode in order to avoid the complex mountainous area caused by the terrain elevation difference, vegetation coverage, and deep valleys, to enhance the identification of potential landslide hazards, and to supplement existing landslide cataloging data. The incidence angles for ascending and descending orbit data are 34.17° and 39.35°, respectively.

**Figure 2:**
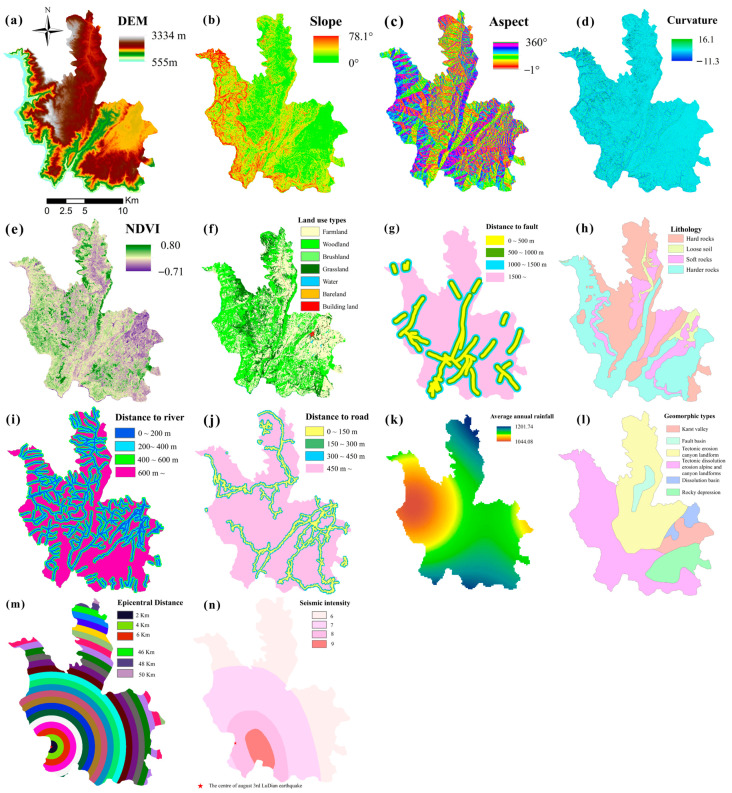
Landslide susceptibility assessment factors. (**a**) DEM. The DEM is the data source for elevation and other topographic factors. (**b**) Slope. Slope describes the degree of slope inclination and influences the landslide stability. (**c**) Aspect. Aspect represents the orientation of the slope and affects the soil moisture and weathering of landslide. (**d**) Curvature. Curvature expresses the rate of change of aspect along a contour. (**e**) NDVI. NDVI is an index that indicates on the growth status and quantity distribution of plants. (**f**) Land use. The Land use affects the soil mechanical properties and hydrological environment. (**g**) Distance to fault. Faults affects the stability of slopes by cutting rocks and soils. (**h**) Lithology. The lithology type affects the landslide development by affecting the shear strength of the slope. (**i**) Distance to river. Rivers affect landslide development because the strength of the rocks and soils are eroded by the rivers. (**j**) Distance to road. The distance to roads is related to human engineering activities. The activities contribute to a change in topography and generally accelerate slope instability. (**k**) Average annual rainfall. Rainfall induces the occurrence of landslide. (**l**) Geomorphic types. The geomorphic types areimportant factors in determining the stability of landslides. (**m**) Epicentral Distance. The epicentral distance map was drawn with the center of the earthquake in Ludian County on 3 August 2014 as the center of the circle and every 2 km as the radius to reflect the relationship between earthquake and landslide in the study area. (**n**) Seismic Intensity. The seismic intensity map indicates the possibility of landslides induced by different earthquake intensities in the study area.

The auxiliary data encompass 0.5 m resolution Google satellite image data, DEM, slope, aspect, and curvature data. These data assisted radar data in inverting the rate of deformation and identifying potential landslide points. Precise orbit determination (POD) data was applied to correct the orbital accuracy of the radar data. Japan Aerospace Exploration Agency (JAXA) ALOS WORLD 3D 30 m resolution DEM (Digital Elevation Model) data was utilized to eliminate the terrain phase effect on surface deformation. As shown in Figure 2, PSO-RF model evaluation factors included precipitation, fault, stratigraphic lithology, river system, land use, normalized difference vegetation index (NDVI), topography, landform, highway network, seismic intensity, and epicenter distance. The specific information of the research data is detailed in Table 1.

## 3. Methodology

**Figure 3:**
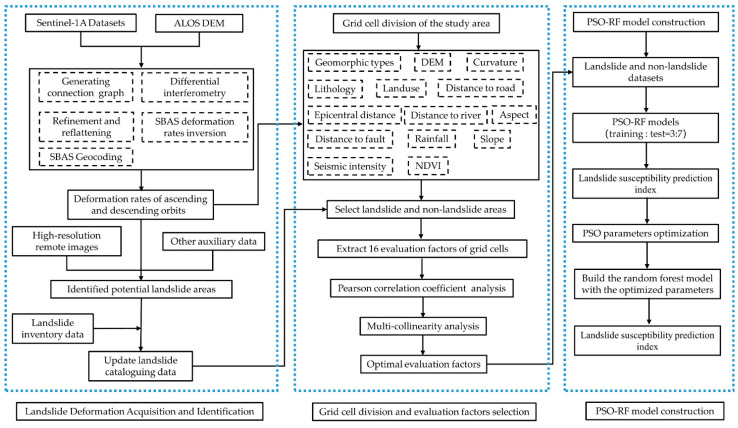
Technical route.

Figure 3 depicts the technical path of this research method, which consists primarily of landslide deformation acquisition and identification, evaluation unit division, evaluation factor selection, and PSO-RF model development.

### 3.1. Landslide Deformation Acquisition and Identification

In the study area, there were 122 existing landslide hazards that were recorded in the landslide cataloging data by December 2021. However, the identification and recording of some potential landslide hazards had not been completed for these hazards. The 61 ascending and 58 descending orbits Sentinel-1A radar datasets were downloaded from the European Space Agency (ESA) website (https://scihub.copernicus.eu/dhus/#/home accessed on 8 December 2021) in order to obtain the deformation data of each landslide hazard in the study area and to identify potential landslide potential hazards. The orbit was corrected using the precise orbit determination (POD) data. The systematic error introduced by orbit error can be effectively eliminated. Simultaneously, the image located in the middle of the time baseline and the frequency center of the Doppler sequence centroid are chosen as the super master image. Throughout the entire processing, the super master image served as the reference image, and all images were registered to it. Afterward, interference processing was conducted on all registered image pairs. To suppress speckle noise more effectively, the range looks and azimuth looks were set to 4:1, and the unwrapping and filtering techniques were Minimum Cost Flow and Goldstein, respectively. After removing unsatisfactory interference data from the interferograms. DEM data were utilized to eliminate the flat land phase and topographic effect in order to generate the time series interference phase. The interference phase of the master and slave image [40,41,42] was as follows:

(1) $$\varphi \approx \varphi_{topo}+\varphi_{def}+\varphi_{atm}+\varphi_{flat}+\varphi_{noise}$$

where $\varphi_{topo}$ is the terrain phase, $\varphi_{def}$ is the deformation phase, $\varphi_{atm}$ is the atmospheric delay phase, $\varphi_{flat}$ is the flat phase, and $\varphi_{noise}$ is the noise phase. The effective deformation data was extracted using phase unwrapping, and the deformation rate was inversed using singular value decomposition (SVD). Finally, the deformations of the time series were geocoded and projected onto the study area. LOS (Line of Sight) direction deformation rate in the study area was obtained using data from ascending and descending orbits. 

Since the rate of deformation in the LOS direction was the projection of the rate of surface deformation in the radar sight line direction, any surface deformation can be expressed by three components: SN (N), EW (E), and vertical (U) [43]. The contribution rate of vertical deformation to the LOS direction of satellite movement is more than 90% regardless of ascending and descending orbits, according to the geometric relationship between radar side-view imaging and the relationship between LOS deformation and surface deformation observed by InSAR [44]. Therefore, the vertical deformation calculation formula presented in this paper was:

(2) $$U_{V}\approx D_{\mathrm{LOS}}/cos\;\theta$$

The study area was located in complex mountainous regions with frequent earthquakes, a large difference in terrain height, dense vegetation, and deep river valleys, as well as severe decoherence issues. This paper considered the use of ascending and descending radar data to obtain accurate and comprehensive monitoring results while avoiding the geometric distortion caused by single orbit data. Additionally, the normalized difference vegetation index (NDVI) was implemented to analyze the vegetation cover in the study area to eliminate the decoherence prompted by vegetation cover.

It is impossible to determine whether a potential landslide hazard exists in the study area based solely on the deformation monitoring results. As a corollary, it is essential to thoroughly assess the deformation range, slope, slope aspect, elevation, curvature, and other data in order to determine whether it is a potential landslide hazard. To prevent inaccurate assessments arising from excessive reliance on surface deformation monitoring results. The cross-validation monitoring results from ascending and descending orbits were chosen in order to confirm the accuracy of identification and the precision of SBAS-InSAR monitoring results, and the field investigation confirmed the precision of potential landslide identification.

### 3.2. Grid Cell Division and Evaluation Factors Selection

The evaluation cell, which can be regular or irregular, is the smallest spatial graph element used in the susceptibility evaluation for landslide hazards [45]. The evaluation of landslide susceptibility necessitates the selection of an appropriate evaluation cell. Five categories can be used to summarize the commonly used evaluation cells: regional cell, slope cell, grid cell, terrain cell, and uniform condition cell. Regional cell is the basis of geographical spatial division and regional policy. Slope cell is the basic unit of the development of landslide disasters, which can obviously reflect the difference of regional geological environment conditions. Grid cell, which divides the study area into regular grids according to a certain size. It is the most widely used assessment cell for landslide susceptibility assessment. Terrain cell refers to the basic cell of land resource survey. It is divided according to the relationship between slope damage and geomorphic environment and is applicable to the assessment of regional landslide susceptibility in small regions and large scales. Unique condition unit is used to obtain several irregular evaluation cells with different sizes by superimposing and analyzing all evaluation factors, which is applicable to a large-scale study area. The best options for mountainous regions with complex terrain are grid cell and slope cell [4]. The slope cell can account for the original natural geographic data, such as the topography and natural slope of the area under study. However, the operation of the slope unit is complicated, subject to subjective factors, and discontinuous, which makes it impossible to ensure accuracy. Despite the fact that the grid cell cannot preserve the original surface morphology of the research area, it is favored by the majority of researchers due to its simple operation, fast calculation speed, timely error correction, and effective visualization of calculation results. Therefore, evaluation cells are divided in this paper using grid cells. Tang et al. [46] proposed an empirical formula for determining the basic size of grid cells:

(3) $$G_{s}=7.5+0.0006S-2.01\times 10^{-9}S^{2}+2.91\times 10^{-15}S^{3}$$

where $G_{s}$ is the grid size, and S is the reciprocal of the basic data scale. By calculation, the size of grid cells in this study was 30 m × 30 m. In consideration of the landslide area, mapping requirements, and other factors, the 60 m × 60 m evaluation grid cell was finally selected for this study. The study area was divided into a total of 412,585 grid cells.

The formation and progression of landslides depend primarily on geological environment conditions and inducing factors. The degree of landslide hazard is closely related to human engineering activities. Ludian county is situated in an active fault zone with frequent earthquakes, making it extremely vulnerable to landslide devastations. Based on previous research, 12 influencing factors, including geological factors, topographic factors, environmental factors, and human engineering activities, were selected [47,48,49]. As evaluation factors, four influencing factors were considered: landslide time series deformation of ascending and descending orbit, seismic intensity released by the Ludian earthquake on 3 August 2014, and epicentral distance with a 2 km radius. Through ArcGIS multi-value extraction to points, 16 evaluation factor attribute values were extracted from the respective grid cells of the landslide area and non-landslide area. The validity and dependability of the 16 evaluation factors were then determined using the Pearson correlation coefficient analysis and multicollinearity analysis using SPSSPRO software.

### 3.3. PSO-RF Model 

Random Forest (RF) is a parallel enhanced machine learning algorithm proposed by Breiman in 2001 [50] that integrates the bagging method and classification regression tree (CART). It extracts multiple samples from the original samples using the Bootstrap resampling method. Modeling of decision trees is performed on each Bootstrap sample. The predictions of multiple decision trees are then combined, and the final prediction result is determined by voting [51].

The user specifies two parameters in the random forest algorithm: first, the number of features (max_features) used in generating the decision tree determines the classification strength of the decision tree in the random forest. The prediction accuracy of the decision tree would suffer if there is an insufficient number of features to reliably classify the data, while an overabundance of features will cause certain boundary values to distort the normal classification result. The second is the number of trees in the random forest (n_estimators). The number of trees in the random forest has a significant impact on its influence. When generating a random forest, if there are too few trees, an underfitting phenomenon may occur, while if there are too many, an overfitting phenomenon may occur. For this reason, this paper presents the particle swarm optimization algorithm to optimize the number of selection trees and the number of features in the random forest, in order to find the best “collocation combination” of these two parameters.

The computational model of the Particle Swarm Optimization (PSO) algorithm [52] is derived from the foraging mode of birds. Initialized with a collection of random particles, the PSO optimization algorithm iteratively determines the optimal solution for the current function. All particles update their position and velocity during each iteration using two “extreme values”. The first “extreme” is the optimal solution found by the particle itself, which is the portion extreme pbest. The other “extreme value” is the optimal solution discovered under the current conditions of the entire particle swarm, specifically the global extreme value gbest (gbest is the best value in pbest).

**Figure 4:**
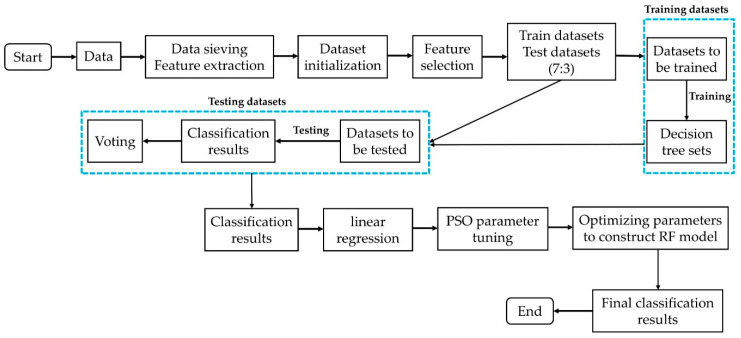
Flow chart of optimizing the parameters of random forest based on PSO.

The algorithm process of the PSO-RF model is as follows: as independent variables, the number of trees in the random forest and the number of constructed random forest features utilized. As the dependent variable, the evaluation index of the model classification result was selected. Assuming that the three are linear, linear regression is applied to the classification results of random forest. To obtain the maximum value, the PSO algorithm was then given the function that was obtained through regression. The number of random forest trees and the number of random forest features with the maximum point were obtained. The random forest is reconstructed, and the data set is classified once more to procure the final classification result, which is, by default, the best classification result. The algorithm building process of PSO-RF model is shown in Figure 4.

Figure 4 shows the construction process of PSO-RF model in detail, and the whole process can be divided into two parts. The first part is the construction of the RF model by continuously adjusting the number of trees and the number of features in the random forest to obtain the set of triples (number of trees, number of features, and the classification accuracy of the random forest with this parameter). In the second part, PSO is used for parameter optimization. The final classification result is obtained by substituting the optimal parameters into the random model and classifying them again.

## 4. Results and Analysis

### 4.1. Surface Deformation Information and Identification of Potential Landslide Hazards 

**Figure 5:**
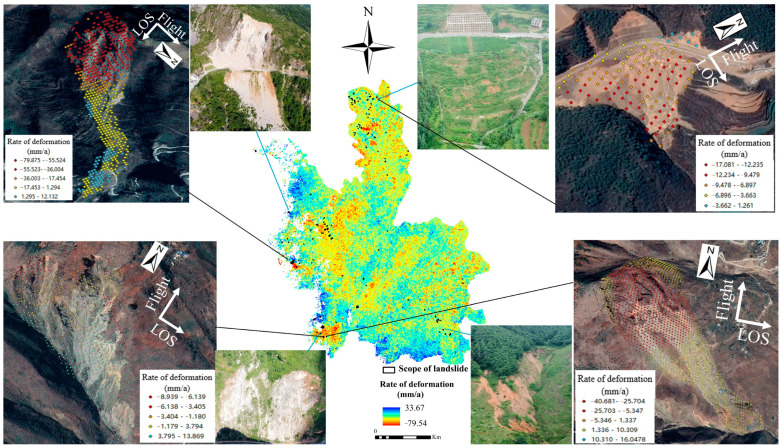
Surface deformation and landslide identification of study area (ascending orbits).

**Figure 6:**
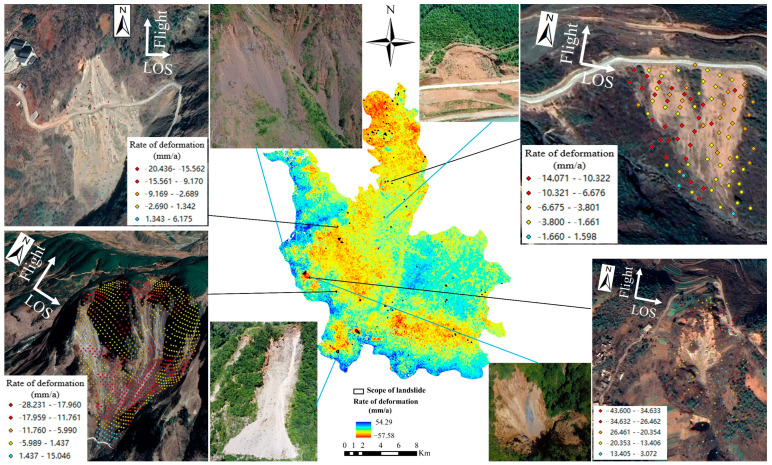
Surface deformation and landslide identification of study area (descending orbits).

The SBAS-InSAR technique is used to process the ascending and descending orbit datasets covering Ludian County. From January 2020 to December 2021, the vertical annual average surface deformation rate field for the study area was obtained. In Figure 5 and Figure 6, moving away from the satellite is represented by the color red, while moving toward the satellite is represented by the color blue. The respective maximum deformation rates are −79.54 mm/a and −57.57 mm/a. The annual mean deformation rate varies between orbits. Due to the collection of two distinct types of orbital data, the satellite’s flight direction is inconsistent. The ascending orbit data satellite generally flies from south to north, with the radar sight line on the right, whereas the descending orbit data satellite flies in the opposite direction. It is feasible to effectively avoid the geometric distortion of SAR imaging caused by single orbit data by combining the processing results of ascending and descending data. This method accurately identifies and monitors potential landslide hazards in the study area.

The ascending and descending orbit deformation rate field, a Google satellite image with a resolution of 0.5 m, the deformation range, DEM, slope, aspect, and the NDVI index were used to determine the characteristics of potential landslide hazards. Potential landslide potential hazards were identified (97 and 122, respectively) in the deformation rate field of ascending and descending orbits. Figure 4 and Figure 5 are depicted as such. In Ludian County, the Yunnan Provincial Department of Natural Resources collected data on 122 landslides by December 2021. Comprehensive processing was performed on the identified potential landslide hazards and the landslide cataloging data. The potential for repeated landslides was eliminated. Finally, 329 landslide hazards were identified in Ludian County. According to the updated distribution map of landslide hazards, the Yunnan Provincial Natural Resources Department delegated subordinate units to conduct field investigation and verification. The results of recognition are consistent with field investigation through superposition with 3D images and field investigation. The solid blue line in Figure 5 and Figure 6 indicates the field map of the field survey. As depicted in the diagram, the landslide traces are evident. The solid black line demonstrates the superposition of the deformation rate in the area of the landslide with the Google 3D image. The superposition diagram clearly demonstrates the presence of landslide traces and activity in the region

**Figure 7:**
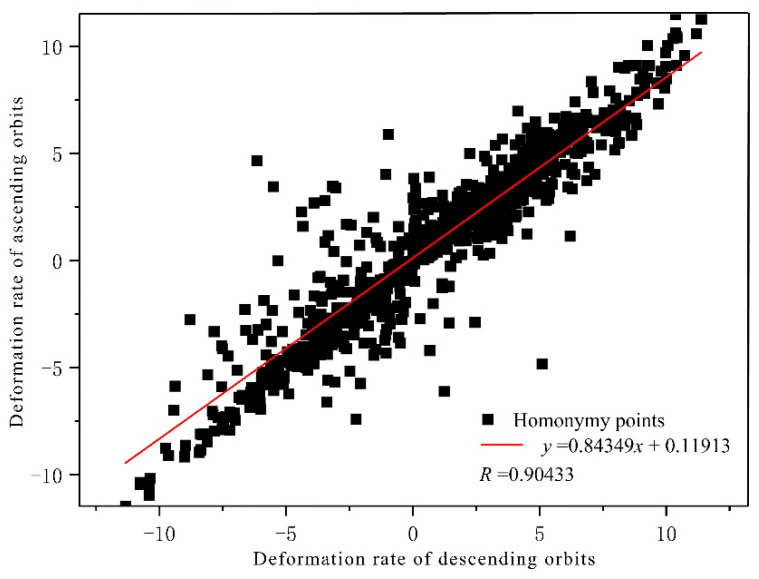
Correlation coefficient diagram of annual average deformation rate of ascending and descending orbits.

Due to the absence of field monitoring data during the same time period, the cross-comparison verification method is used to validate the accuracy of SBAS-InSAR monitoring results. SBAS-InSAR technology was adopted to obtain the deformation rate field of the ascending and descending orbits, from which 1182 corresponding points were selected at random. The ascending average annual settling rate served as the vertical axis of the correlation coefficient diagram, while the descending average annual settling rate served as the horizontal axis. As depicted in Figure 7, the correlation coefficient R between the two variables is 0.90433, while $R^{2}$ is 0.81781. It demonstrates that the monitoring results presented in this paper are highly consistent and correlated.

### 4.2. Evaluation Factor Analysis

Geological environment variations are frequently the cause of landslides. However, the geological environment is a system that is extremely complicated and challenging to explain. This system is influenced by a wide variety of factors, including both internal and external factors. Consequently, the key to evaluating landslide susceptibility lies in determining these influence factors. All of the evaluation factors chosen for this paper can contribute to landslide formation to some degree. In theory, these influencing factors can be used as evaluation factors and incorporated into the evaluation model for prediction, but in practice, there may be a strong correlation or multicollinearity among evaluation factors. If these variables are incorporated into the model, issues such as sluggish model execution, model complexity, and model overfitting may arise, influencing the evaluation results of the model. Therefore, it is necessary to analyze each factor prior to establishing the model in order to simplify the model and enhance its performance so as to ensure the accuracy of the evaluation results.

In this article, the correlation coefficient between evaluation factors is calculated using Pearson’s correlation coefficient, which is widely used and simple to implement. In this study, a Pearson correlation coefficient of less than 0.5 indicates that there is either a weak correlation or almost no correlation. The correlation between variables with Pearson coefficients between 0.5 and 0.8 is moderate, whereas correlations greater than 0.8 are strong. The mathematical analysis software SPSSPRO was used to select 2632 sample points, 1316 of which were landslide sample points and 1316 of which were not. Table 2 displays the correlation coefficient between the final evaluation factors as determined by the correlation analysis. According to Table 2, the Pearson correlation coefficient between DEM and the rate of surface deformation of descending orbits in the study region was 0.587. The Pearson correlation coefficient between the epicentral distance and seismic intensity map for the 3 August 2014 earthquake in Ludian County was −0.790. The correlation between the two Pearson correlation coefficients was moderate. Due to the complexity of the evaluation factors for landslide susceptibility, there is a strong correlation between the variables. Factors with a strong correlation should not be excluded based solely on the results of a Pearson correlation analysis; rather, the results of a factor multicollinearity analysis should be incorporated.

In linear regression analysis, multicollinearity analysis is a statistical evaluation technique used to determine whether there is a high linear correlation between independent variables. In general, the variance inflation factor (VIF) and tolerance (TOL) are utilized to assess the multicollinearity between factors. If TOL is less than 0.5 and VIF is greater than 2, a strong multicollinearity among factors is indicated. A problem with multicollinearity would not exist otherwise. After filtering, multiple linear analysis of each index factor was performed in this paper using SPSS 26 mathematical analysis software. According to Table 3, the VIF value and TOL value of the epicenter distance of the earthquake that occurred in Ludian County on 3 August 2014, are 4.213 and 0.237, respectively. The epicenter distance factor generated with the 2 km radius of the earthquake center in Ludian County on 3 August 2014 was eliminated, and the remaining 15 evaluation factors were retained as the final evaluation factors of landslide susceptibility in this study area. The retained factors were validated through correlation and multicollinearity analyses. The results were presented in Table 4 and Table 5. Table 4 and Table 5 demonstrate that there is neither a moderate nor a high correlation between the retained evaluation factors nor is there multicollinearity, thereby validating the reliability and validity of the chosen evaluation factors.

### 4.3. Landslide Susceptibility Model Construction and Evaluation Result Analysis

#### 4.3.1. PSO-RF Model Construction of the Ludian County

In this paper, 329 potential landslide hazards in the study area were identified by combining the deformation results of SBAS-InSAR monitoring and the landslide cataloging data of the Yunnan Provincial Natural Resources Department. The identified landslide potential hazards and non-landslide potential hazards are quantified and processed. The landslide susceptibility evaluation is transformed into a dichotomous problem by the use of “1” to represent the high susceptibility area and “0” to represent the low susceptibility area. The study area was divided using 60 m × 60 m grid cells, and 2722 landslide (1361 grid cells) and non-landslide (1361 grid cells) grid cells in the study area were used to construct the PSO-RF model. If the basic dataset is improperly partitioned into the training set and the test set, the predictive accuracy of the model will be compromised. In the process of model construction, the ratio of training dataset to test dataset was set to 7:3 based on relevant literature research [52,53].

**Figure 8:**
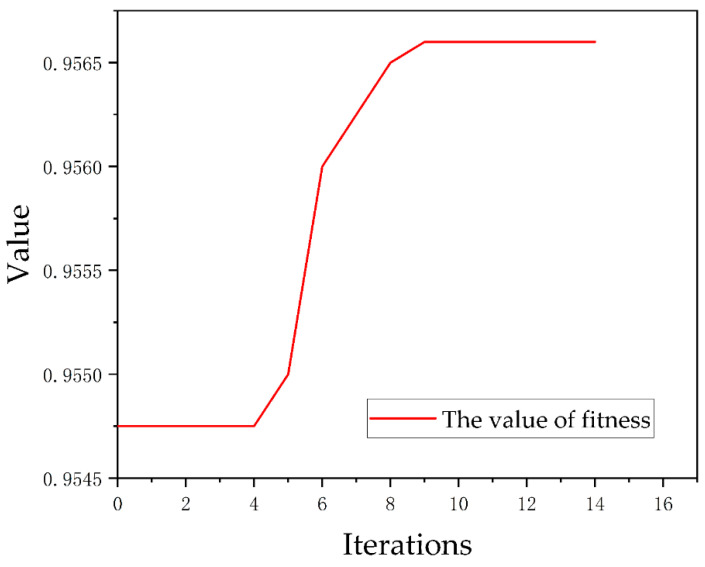
The variation of function maxima with the number of iterations.

The PSO-RF model was constructed using Python language through the Scikit-Learn framework, and the model was hyperparametrically tuned by the PSO algorithm. In the process of PSO algorithm optimization, the variations of the function maxima with the number of iterations are shown in Figure 8. As can be seen from Figure 8, when PSO was optimizing the parameters, the maximum value can be obtained in the ninth iteration, and the maximum value remained constant during the continuous iterations. Therefore, PSO is successful in optimizing parameters. After tuning, the number of decision trees of the PSO-RF model was 52, the maximum depth was 20, the minimum number of node sample segmentation was 2, and the minimum number of sample leaves and the maximum number of features were 1. The optimal parameters of the PSO-RF model were saved, and the selected 15 evaluation factors were input into the PSO-RF model to calculate the landslide susceptibility index of each grid cell in the study area. The natural break point grading method in the ArcGIS 10.2 software platform combined with expert participation was used to grade the landslide susceptibility index, and the landslide hazard susceptibility zoning map was drawn to evaluate the landslide susceptibility of the study area.

#### 4.3.2. Analysis of Evaluation Results

**Figure 9:**
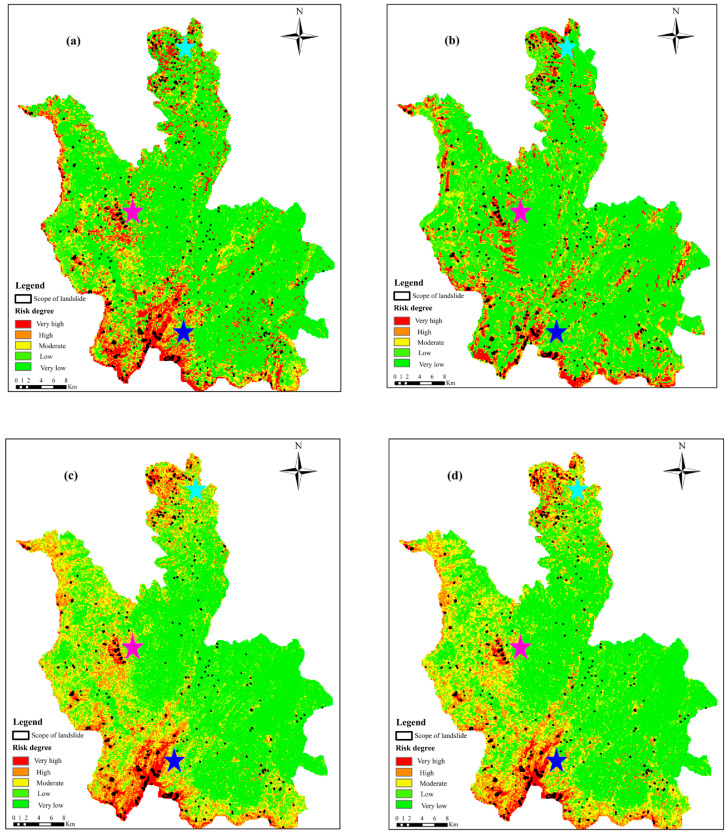
Predicted landslide susceptibility for the county of Ludian. (**a**–**d**) Back Propagation algorithm, Support vector machines, Random Forest, and PSO-RF models, respectively. The blue star represents Longtoushan Town, the earthquake’s epicenter on 3 August 2014. The position of the magenta star is Shuimo Town. The cyan star represents the town of Xinjie.

First, the evaluation factor data of the entire study area were extracted from ArcGIS 10.2 software and input into the trained model to obtain the classification results and landslide susceptibility index. Then, according to the landslide susceptibility classification method described in the literature [31,32], the landslide susceptibility in the study area was divided into five categories. The natural break point grading method combined with the participation of experts was used to carry out the susceptibility classification treatment, which was divided into (0, −0.15], (−0.15, 0.25], (0.25, 0.65], (0.65, 0.85], and (0.85, 1], which correspond to extremely low susceptibility area, low susceptibility area, medium susceptibility area, high susceptibility area, and extremely high susceptibility area. The landslide susceptibility probabilities of some grid cells calculated by the four model are shown in Table 6. Finally, leveraging the reclassification function of ArcGIS 10.2 software, a zoning map of landslide susceptibility evaluation in the study area supported by SABS-InSAR technology and the PSO-RF model, was created. Four models, including the Back Propagation (BP) algorithm, Support vector machines (SVM), and Random Forest (RF), were selected in order to validate the reliability and accuracy of the PSO-RF evaluation model. The performance of the PSO-RF model is validated by comparing the evaluation results, and the results are depicted in Figure 9.

By comparing the evaluation results of landslide susceptibility calculated by the four models and the distribution law of landslide hazards (Figure 9), it can be concluded that the zoning results are characterized by the following:

(1) The high to extremely high landslide hazard zone is primarily located along the territory’s border. It extends from the northwest to the southeast. Near Longtoushan town, the north bank of the Niulanjiang River is particularly prone to landslides (near the blue star). This area covers an area of 602.01 km^2^. The geomorphic type is mainly tectonic erosion, deep cut mountain gorge topography, and gully development. The altitude is 500~3300 m, and the terrain slope is 20~50°. It is mainly medium-steep slope to steep slope and about 25% forest coverage. The outcrop layer is complete with great lithologic changes, mainly including Permian (P_1__–2_), Ordovician (O_1__–__3_), Cambrian ($\in_{1–3}$) sand mudstone, limestone interbedding, and basalt. The geotechnical engineering property belongs to soft rock and semi-hard to hard interphase rock group. There are three northeast faults through fold development, rock fragmentation, strong weathering, and geological disaster development; it is a strong geological disaster activity area.
(2) Near Shuimo Town (magenta asterisk) and Xinjie Town (cyan asterisk) are two additional high to extremely high landslide hazard areas. Landslides in these two areas are more developed. The geomorphic type of Shuimo Town (magenta asterisk) is mainly tectonic denudation in the high mountain valley terrain with an altitude of 1000~2500 m, a terrain slope of 15~35°, mainly gentle to medium steep slopes, and a forest coverage rate of about 25%. The exposed strata are mainly composed of Triassic system (T1f) and Permian system (P_1_q+m, P_2_β) limestone and basalt, followed by Ordovician (O_1–3_) and Cambrian (∈_2–3_) limestone interbedded with sand mudstone and shale, belonging to hard and soft rock formation.
(3) Xinjie Town (cyan asterisk) is located in the northern region, with an area of 165.77 km^2^. The geomorphological type is mainly tectonic erosion alpine terrain, with an altitude of 2400~2950 m, and a terrain slope of 5~25°, mainly with a gentle slope and a forest coverage of about 20%. The exposed stratum is mainly basalt and diagenetic (P_1–2_) limestone, and the geotechnical properties are a soft rock to hard rock group. Due to the weathering and fragmentation of basalt, when rainfall occurs, the surface soil slides, resulting in a large number of landslides and geological hazards.
(4) Compared to the other three models, the random forest model based on particle swarm optimization has fewer landslides distributed in the low-prone area and more landslides distributed in the extremely high-prone area, which is practically advantageous.

## 5. Discussion

### 5.1. Model Precision Analysis

**Figure 10:**
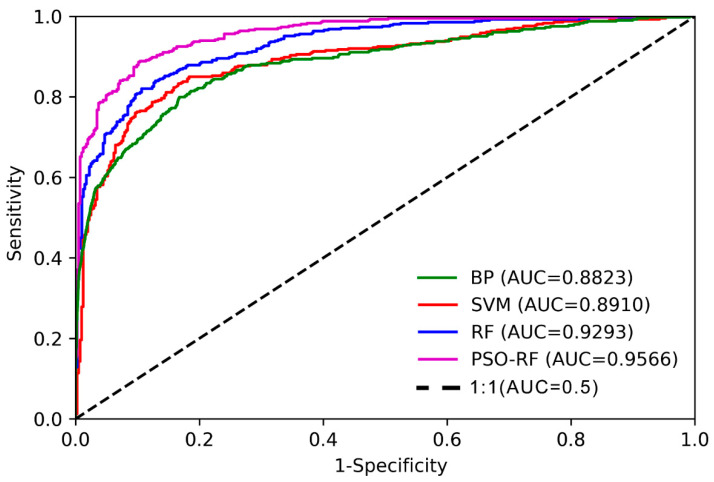
ROC curve and AUC value of the evaluation models.

The purpose of accuracy evaluation is to assess the predictive performance of a model. The comparison of the classification results with the actual results served as an example of the model performance (how accurate the prediction is). From a qualitative perspective, the outcomes of landslide susceptibility prediction are shown in Figure 9, demonstrating that the distribution patterns of landslide susceptibility predicted by the four models developed in this study are identical in Ludian County, proving the applicability and reliability of machine learning models in landslide susceptibility prediction. The receiver-operating characteristic (ROC) curve, the area under the curve (AUC), and the accuracy (ACC) are utilized for quantitative evaluation. The closer the ROC curve is to the upper left, the better it is, whereas the closer it is to the lower right, the worse it is, and a curve below the reference line indicates that the model is completely unusable. The AUC value ranges from 0 to 1. When the value is higher, it indicates that the model is more accurate. On the basis of AUC values, model accuracy levels can be categorized as follows: 0.5 to 0.6 (poor), 0.6 to 0.7 (moderate), 0.7 to 0.8 (good), 0.8 to 0.9 (excellent), and 0.9 to 1.0 (near perfect) [53]. As shown in Figure 10, the performance of the BP, SVM, and RF models for assessing landslide susceptibility in Ludian County is above excellent, with the random forest model performing the best, followed by the SVM model, and then the BP model. To further quantify the performance of the prediction model, the ACC value was selected to evaluate the model’s performance. The ACC values were computed using a confusion matrix that reveals the relationship between the model’s predicted and actual results. Table 7 displays the results of the calculations, which revealed that the random forest model had the best performance among the single models, with an ACC value of 0.8531, which was 2.57 and 2.20 percentage points higher than BP and SVM, respectively. Using the fast global optimization search function of the PSO algorithm, the particle swarm algorithm optimized the number of decision trees (n_estimators) and the number of random forest features (max_features) to choose the best random forest model. The AUC and ACC values of the PSO-RF model were 0.9567 and 0.8874, outperforming the random forest model by 2.74 and 3.43 percentage points for the same set of input features of the landslide prediction model. The results indicated that the PSO-RF model indicates a near-perfect prediction performance in predicting the landslide susceptibility in complex mountainous regions and was more applicable to the evaluation of landslide susceptibility prediction in this study area than the other three models.

### 5.2. Comparison with the Grading Evaluation Factor

According to the reviewed literature [47,54], first, we graded the input variables (15 evaluation factors) and calculated the frequency ratios for each factor after grading. The results of the grading factors and frequency ratio calculation are shown in Table 8. Finally, the frequency ratio of the evaluation factor was input into the PSO-RF model constructed in this paper and the other three machine learning models (BP, SVM, and RF) to predict the landslide probability of 412,585 grid cells in the study area. The landslide susceptibility of the study area was graded based on the probabilities of each grid point using the natural breakpoint method in the ArcGIS 10.2 software platform. During the experiments, the input variables were graded based on the literature [53]. For example, the slope directions varying from 0 to 360° were divided into eight directions, i.e., north, northeast, east, southeast, south, southwest, west, and northwest, values close to 360° and 0° were combined as the north direction, and the frequency ratios were calculated for each direction. In the modeling process, the model parameters were the same as the prediction model when the input variables are not graded. Considering the graded input variables, the predicted results of the three models, BP, RF, and PSO-RF, were consistent with the trend of ungraded input variables. However, the SVM model was used to input the graded variables. Compared with the prediction results of the input variables without grading, the prediction results of the northeast, east, and southeast directions of the study area were very poor. To further describe the accuracy of the prediction results, we counted the AUC and ACC values for the four methods, as shown in Table 9. It can be seen from Table 9 that, after grading the input variables, the AUC and ACC values were lower than those of the ungraded input variables. The reason may be that the study area was located in the north bank of Niulan River with huge terrain elevation differences, crisscross canyons, active fault zones, strong tectonic movement, and frequent earthquakes, which make the rock and soil mass in the region broken. Under the influence of special geological conditions, rainfall, and earthquake, the randomness of landslide occurrences is very large. If the input variables are graded, the effect of some environmental factors will be ignored, which will reduce the prediction accuracy. Therefore, this paper chose to directly input evaluation factors for the landslide susceptibility evaluation.

### 5.3. Landslide Susceptibility Evaluation Model Analysis

The study of the landslide susceptibility evaluation yielded a large number of successful examples from both domestic and international researchers. However, there are still drawbacks, such as the inability to detect landslide activity, the lack of timely landslide disaster data sources, and the requirement of a large number of experts to participate in statistics. Targeting the issues of slow updates and ineffectiveness of data sources for landslide disasters, in this paper, the SBAS-InSAR technology, a Google satellite image with a resolution of 0.5 m, and other auxiliary data were used to identify landslide disasters in complex mountainous regions with frequent earthquakes, deep valleys, and high topographic elevation. The surface deformation rate was inversed by calculating the phase variation of the ascending and descending orbit radar images. Resultantly, the active situation of landslides and potential landslide hazards could be more accurately identified. The accuracy of the InSAR recognition results could be enhanced by incorporating a Google satellite image with a resolution of 0.5 m and auxiliary data. This paper proposed using a PSO-RF model to predict the susceptibility of landslides in an effort to mitigate the disadvantage of requiring a large number of experts to participate in statistics. During the modeling procedure, the susceptibility index of the grid cells in the study area was predicted by inputting various grid cell learning evaluation factors. This effectively avoided a large number of expert statistics and reduced the manual participation error in the calculations, thereby improving the accuracy of the evaluation model. This paper integrated the SBAS-InSAR technique to obtain the surface deformation rate under different orbit (ascending and descending orbit) operations of the satellite to address the problem that landslide activity cannot be detected. This method was used to identify existing landslides and potential landslides in the study area, thereby increasing the efficacy of the data source for landslide disasters. Due to the relatively high weight of the evaluation factors, some stable landslide points without deformation were prevented from being evaluated as extremely high or high areas.

Compared with traditional landslide hazard survey techniques, the method proposed in this paper can quickly update landslide data sources, detect landslide activity, and effectively avoid a large number of statistical calculations with experts. A landslide susceptibility evaluation in complex mountainous areas can be quickly carried out. However, there are some shortcomings in the selected evaluation factors. For example, due to the lack of detailed formation of the lithology data during the experiment, the formation lithology was simply divided into four categories: hard rocks, loose soil, soft rocks, and harder rocks. Different formation lithology has different shear strengths, and the possibilities of landslides are not same. In the next study, we will obtain more detailed evaluation factors data and explore the general applicability of the model.

## 6. Conclusions

By analyzing the problems of the existing landslide hazard susceptibility evaluation model, such as poor effectiveness and inaccuracy of the landslide hazard data and the need for experts to participate in the calculation of a large number of evaluation factor weight classification statistics, in this paper, a combined SBAS-InSAR and PSO-RF algorithm was proposed to evaluate the susceptibility of landslide disasters in complex mountainous regions. In the experiment, 61 ascending and 58 descending orbits Sentinel-1A radar datasets were used to invert the times-series deformation of Ludian County from January 2020 to December 2021. Then, potential landslide hazards in the study area were identified with the support of auxiliary data, such as high-resolution optical remote sensing, DEM, and the slope, and the landslide cataloguing data sources were updated after the identification results were verified in the field. Finally, the PSO-RF model was constructed to evaluate the landslide susceptibility of the study area. Based on the study, the following conclusions can be drawn:

(1) Compared to traditional landslide disaster survey techniques (such as field investigation, GNSS monitoring, etc.), the SBAS-InSAR technology can quickly determine the surface deformation of the study area. The technique identified 97 and 122 potential landslide hazards in the ascending and descending deformation rate field, respectively, updating the existing landslide cataloging data to 329.
(2) Through analysis and verification, the ascending and descending orbit deformation rates obtained by the SBAS-InSAR technique can be used as a significant factor in the classification of landslide susceptibility.
(3) By analyzing real landslide and non-landslide data, the performances of the PSO-RF algorithm and three other machine learning algorithms, BP (back propagation), SVM (support vector machines), and RF (random forest) algorithms, were compared. The results showed that the PSO-RF model proposed in this paper had the best performance and evaluation results. The area under the curve (AUC) value and the accuracy (ACC) of the PSO-RF algorithm were 0.9567 and 0.8874, which were higher than those of the BP (0.8823 and 0.8274), SVM (0.8910 and 0.8311), and RF (0.9293 and 0.8531), respectively.
(4) The method proposed in this paper, on the one hand, effectively identified the deforming and potential landslide hazards in the study area, quickly updated the landslide data source, and solved the problems of poor effectiveness and uncertainty of the existing landslide hazard data source. On the other hand, the disadvantage of the traditional landslide susceptibility evaluation model, which requires weight calculation and statistical classification, is prevented by the PSO-RF model developed in this paper. In terms of prediction, it avoided a significant amount of manual expert decision-making. It can serve as a useful reference for future disaster prevention and reduction decisions made by government departments.

Landslide disasters are characterized by a complex mechanism of development and strong suddenness. Even though the impact of seismic intensity on landslide susceptibility in complex mountainous regions is considered in this study, there are still many issues that require further research. For instance, the more important aspects of landslide risk management should be investigated. Investigating the process of landslide formation, in order to explore the possibility of developing a more optimized landslide intelligent model, both landslides precipitated by rainfall and earthquakes are analyzed independently. In future research, we intend to continue conducting pertinent research premised on the aforementioned considerations.

## Figures and Tables

**Table 1:** List of data sources.

Data Name	Data Scale	Data Phase	Data Source
Sentinel-1A	5 m × 20 m	January 2020–December 2021	European Space Agency
Precise Orbit Determination	-	January 2020–December 2021	European Space Agency
Google satellite image	0.5 m	January 2020–December 2021	Google earth sofeware
DEM	30 m	2021	Japan Aerospace Exploration Agency
Slope, aspect and curvature	30 m	2021	Obtained from DEM processing
Rainfall	30 m	January 2020–December 2021	China Meteorological Administration
Faultage	1:10,000	-	Department of Natural Resources of Yunnan Province, China
River system	1:10,000	2021	Nation Geomatics Center of China
Lithology	1:10,000	2010	Global stratigraphic lithology database
Landuse and NDVI	30 m	2021	Resource and Environment Science and Data Center
Geomorphic types	1:10,000	2010	Geographical Information Monitoring Cloud Platform of China
Road Network	1:10,000	2021	Bigmap sofeware
Seismic Intensity	1:10,000	2014	China Earthquake Administration
Epicentral Distance	-	2014	China Earthquake Administration

**Table 2:** Pearson correlation coefficient of landslide susceptibility assessment factors.

	1	2	3	4	5	6	7	8	9	10	11	12	13	14	15	16
1	1.000															
2	−0.138	1.000														
3	−0.121	−0.107	1.000													
4	−0.279	0.015	0.102	1.000												
5	0.332	−0.073	−0.008	−0.044	1.000											
6	−0.458	0.093	0.290	0.315	−0.203	1.000										
7	0.036	0.115	0.074	−0.039	0.144	0.098	1.000									
8	0.168	0.073	−0.357	−0.030	−0.084	−0.085	−0.109	1.000								
9	−0.072	0.007	0.054	−0.089	−0.176	0.105	−0.059	0.085	1.000							
10	−0.587	0.156	0.055	0.155	−0.231	0.283	0.076	−0.042	−0.058	1.000						
11	−0.151	0.199	−0.024	−0.128	−0.060	0.016	0.192	−0.010	−0.050	0.388	1.000					
12	0.056	0.106	−0.037	−0.028	−0.079	0.035	−0.032	0.047	0.036	−0.062	−0.063	1.000				
13	−0.480	0.009	0.183	0.250	−0.362	0.437	−0.015	−0.127	0.099	0.449	0.081	−0.021	1.000			
14	0.134	0.001	−0.038	−0.033	0.013	0.014	0.000	0.036	−0.004	−0.047	0.004	0.034	−0.025	1.000		
15	0.493	−0.035	−0.109	−0.270	0.378	−0.439	0.132	−0.034	−0.183	−0.329	0.074	−0.055	−0.790	0.051	1.000	
16	−0.160	0.059	−0.156	−0.076	−0.116	−0.015	0.037	0.087	−0.070	0.365	0.307	0.028	0.174	0.025	0.099	1.000

Note: 1: DEM, 2: Aspect, 3: Land use, 4: Geomorphic, 5: Lithology, 6: Slope, 7: Distance to fault, 8: NDVI, 9: Distance to road, 10: The rate of deformation (descending), 11: The rate of deformation (ascending), 12: Distance to river, 13: Seismic Intensity, 14: Curvature, 15: Epicentral distance, and 16: Average annual rainfall.

**Table 3:** Multicollinearity analysis of landslide susceptibility assessment factors.

No.	Factor	TOL	VIF	No	Factor	TOL	VIF
1	DEM	0.546	1.842	9	Dis_Road	0.900	1.112
2	Aspect	0.895	1.118	10	Descending	0.583	1.870
3	landuse	0.763	1.310	11	Ascending	0.731	1.369
4	Geomorphic	0.810	1.235	12	Dis_river	0.941	1.063
5	Lithology	0.767	1.303	13	Seismic Intensity	0.551	1.878
6	Slope	0.623	1.605	14	Curvature	0.968	1.033
7	Dis_Fault	0.876	1.141	15	Epicentral Distance	0.237	4.213
8	NDVI	0.743	1.347	16	Rainfall	0.637	1.569

**Table 4:** Pearson correlation coefficient of retained landslide susceptibility assessment factors.

	1	2	3	4	5	6	7	8	9	10	11	12	13	14	15
1	1.000														
2	−0.138	1.000													
3	−0.121	−0.107	1.000												
4	−0.279	0.015	0.102	1.000											
5	0.332	−0.073	−0.008	−0.044	1.000										
6	−0.458	0.093	0.290	0.315	−0.203	1.000									
7	0.036	0.115	0.074	−0.039	0.144	0.098	1.000								
8	0.168	0.073	−0.357	−0.030	−0.084	−0.085	−0.109	1.000							
9	−0.072	0.007	0.054	−0.089	−0.176	0.105	−0.059	0.085	1.000						
10	−0.487	0.156	0.055	0.155	−0.231	0.283	0.076	−0.042	−0.058	1.000					
11	−0.151	0.199	−0.024	−0.128	−0.060	0.016	0.192	−0.010	−0.050	0.388	1.000				
12	0.056	0.106	−0.037	−0.028	−0.079	0.035	−0.032	0.047	0.036	−0.062	−0.063	1.000			
13	−0.480	0.009	0.183	0.250	−0.362	0.437	−0.015	−0.127	0.099	0.449	0.081	−0.021	1.000		
14	0.134	0.001	−0.038	−0.033	0.013	0.014	0.000	0.036	−0.004	−0.047	0.004	0.034	−0.025	1.000	
15	−0.160	0.059	−0.156	−0.076	−0.116	−0.015	0.037	0.087	−0.070	0.365	0.307	0.028	0.174	0.025	1.000

Note: 1: DEM, 2: Aspect, 3: Land use, 4: Geomorphic, 5: Lithology, 6: Slope, 7: Distance to fault, 8: NDVI, 9: Distance to road, 10: The rate of deformation (descending), 11: The rate of deformation (ascending), 12: Distance to river, 13: Seismic Intensity, 14: Curvature, and 15: Average annual rainfall.

**Table 5:** Multicollinearity analysis of retained landslide susceptibility assessment factors.

No.	Factor	TOL	VIF	No	Factor	TOL	VIF
1	DEM	0.573	1.716	9	Dis_Road	0.907	1.102
2	Aspect	0.895	1.117	10	Descending	0.583	1.669
3	landuse	0.768	1.301	11	Ascending	0.742	1.348
4	Geomorphic	0.810	1.235	12	Dis_river	0.957	1.045
5	Lithology	0.771	1.297	13	Seismic Intensity	0.598	1.673
6	Slope	0.626	1.596	14	Curvature	0.968	1.033
7	Dis_Fault	0.892	1.121	15	Rainfall	0.637	1.569
8	NDVI	0.798	1.253				

**Table 6:** The landslide susceptibility probabilities of some grid cells calculated by the four models.

No.	BP	SVM	RF	PSO-RF
1	0.93469	0.78903	0.56324	0.63250
2	0.93444	0.81064	0.58424	0.63250
3	0.87181	0.79045	0.48907	0.58150
4	0.77966	0.82517	0.43870	0.50118
5	0.99260	0.83340	0.68815	0.72375
6	0.99181	0.79314	0.58716	0.66074
7	0.98918	0.72790	0.56027	0.67305
8	0.99154	0.37857	0.63954	0.68731
9	0.89016	0.71268	0.57285	0.51959
10	0.66734	0.69062	0.44569	0.41138
11	0.84529	0.69656	0.53303	0.53000
12	0.23069	0.85213	0.36400	0.30197
13	0.27658	0.75960	0.36323	0.28322
14	0.40291	0.84241	0.58693	0.62034
15	0.15284	0.85439	0.42343	0.48093
16	0.13227	0.85310	0.50376	0.58434
17	0.93277	0.81977	0.71543	0.70960
18	0.91254	0.80852	0.67964	0.72582
19	0.86692	0.76548	0.52227	0.66591
20	0.77794	0.80081	0.57000	0.54559

**Table 7:** AUC (Area Under the Curve) values and accuracy of the assessment model.

Prediction Performance	Prediction Models			
	BP	SVM	RF	PSO-RF
True positive	335	337	358	364
True negative	341	342	339	361
False positive	73	71	74	52
False negative	68	67	46	40
ACC	0.8274	0.8311	0.8531	0.8874
AUC	0.8823	0.8910	0.9293	0.9567

**Table 8:** Landslide susceptibility evaluation factors grading system.

Factors	Classification	Frequency Ratio	Factors	Classification	Frequency Ratio
Distance to road (m)	0~150	0.9821	Aspect (°)	north	0.8253
	150~300	1.4254		northeast	0.9910
	300~450	1.0858		east	1.2516
	>450	0.9455		southeast	2.3130
Distance to river (m)	0~200	0.6529		south	5.1216
				southwest	1.9307
	200~400	0.9905		west	0.8519
	400~600	1.0744		northwest	0.7793
	>600	1.1343	Curvature	<0	3.4454
Distance to fault (m)	0~500	1.2648		l	0.8214
	500~1000	0.9558		>0	2.4045
	1000~1500	1.2772	Surface deformation of the descending orbits (mm/a)	<−25	0.0000
	>1500	0.8955		−25~0	0.4074
Land use types	farmland	0.1772		0~25	1.3936
	woodland	0.3771		>25	7.2444
	bush	1.8004	Surface deformation of the ascending orbits (mm/a)	<−25	−1.1344
	grass	4.2020		−25~0	−0.1812
	Bare land	3.5963		0~25	0.0796
Average annual rainfall (mm)	<1050	1.0267		>25	0.2907
	1050~1100	0.9947		<−25	−1.2339
	1100~1150	1.0045	Lithology	harder rocks	1.0508
	1150~1200	0.9924		hard rocks	1.0322
	>1200	0.0000		Soft rocks	1.9544
DEM (m)	<1000	2.4147		Loose soil	−2.1400
	1000~1500	1.0372	NDVI	<0.15	3.0372
	1500~2000	0.7658		0.15–0.24	1.7648
	2000~2500	0.0455		0.24–0.32	1.6286
	>2500	0.0689		0.32–0.42	0.7692
Slope (°)	<18	0.2480		>0.42	0.0830
	18~25	0.7564	Seismic Intensity	VI	0.3840
	25~45	2.6952		VII	0.3066
	45–51	5.5447		VIII	1.3919
	>51	7.7629		IX	10.2688
Geomorphic types	rocky depression	0.2234			
	dissolution basin	0.0692			
	karst valley	0.0518			
	fault basin	0.0000			
	tectonic dissolution erosion alphineand canyon landform	2.0551			
	Tectonic erosion canyon landform	0.3077			

**Table 9:** AUC value and accuracy of the prediction results with ungrading and grading evaluation factors.

Prediction Performance	Ungrading Evaluation Factors				Grading Evaluation Factors			
	BP	SVM	RF	PSO-RF	BP	SVM	RF	PSO-RF
ACC	0.8274	0.8311	0.8531	0.8874	0.8016	0.7924	0.8153	0.8519
AUC	0.8823	0.8910	0.9293	0.9567	0.8825	0.8585	0.9135	0.9350

## Data Availability

The data presented in this study are available upon request from the corresponding author.

## References

[1] Xu Q., Lu H.Y., Li W.L., Dong X.J., Guo C. (2022). Types of Potential Landslide and Corresponding Identification Technologies. Geom. Inf. Sci. Wuhan Univ..

[2] Zhang K., Wang S., Bao H.J., Zhao X.M. (2019). Characteristics and influencing factors of rainfall-induced landslide and debris flow hazards in Shaanxi Province, China. Nat. Hazards Earth Sys..

[3] Lin Q., Wang Y. (2018). Spatial and temporal analysis of a fatal landslide inventory in China from 1950 to 2016. Landslides.

[4] Zhou D.Y., Zuo X.Q., Xi W.F., Xiao B., You H. (2021). Combined SBAS-InSAR and PSO-BP algorithm for evaluating the risk of geological disasters in alpine valley regions. Trans. Chin. Soc. Agric. Eng..

[5] Guzzetti F., Carrara A., Cardinali M., Reichenbach P. (1999). Landslide hazard evaluation: A review of current techniques and their application in a multi-scale study, Central Italy. Geomorphology.

[6] Zhu A.X., Wang R., Qiao J., Qin C.Z., Chen Y., Liu J., Du F., Lin Y., Zhu T. (2014). An expert knowledge-based approach to landslide susceptibility mapping using GIS and fuzzy logic. Geomorphology.

[7] Ghosh J.K., Bhattacharya D. (2009). Knowledge-Based Landslide Susceptibility Zonation System. J. Comput. Civ. Eng..

[8] Kayastha P., Dhital M.R., De Smedt F. (2013). Application of the analytical hierarchy process (AHP) for landslide susceptibility mapping: A case study from the Tinau watershed, west Nepal. Comput. Geosci..

[9] Pourghasemi H.R., Pradhan B., Gokceoglu C. (2012). Application of fuzzy logic and analytical hierarchy process (AHP) to landslide susceptibility mapping at Haraz watershed, Iran. Nat. Hazards.

[10] Myronidis D., Papageorgiou C., Theophanous S. (2015). Landslide susceptibility mapping based on landslide history and analytic hierarchy process (AHP). Nat. Hazards.

[11] Wubalem A., Meten M. (2020). Landslide susceptibility mapping using information value and logistic regression models in Goncha Siso Eneses area, northwestern Ethiopia. SN Appl. Sci..

[12] He H., Hu D., Sun Q., Zhu L., Liu Y. (2019). A Landslide Susceptibility Assessment Method Based on GIS Technology and an AHP-Weighted Information Content Method: A Case Study of Southern Anhui, China. ISPRS Int. J. Geo-Inf..

[13] Yu C., Chen J. (2020). Application of a GIS-Based Slope Unit Method for Landslide Susceptibility Mapping in Helong City: Comparative Assessment of ICM, AHP, and RF Model. Symmetry.

[14] Zhang S., Li C., Peng J., Peng D., Xu Q., Zhang Q., Bate B. (2021). GIS-based soil planar slide susceptibility mapping using logistic regression and neural networks: A typical red mudstone area in southwest China. Geomat. Nat. Hazards Risk.

[15] Li H., Chen Y., Deng S., Chen M., Fang T., Tan H. (2019). Eigenvector Spatial Filtering-Based Logistic Regression for Landslide Susceptibility Assessment. ISPRS Int. J. Geo-Inf..

[16] Xing X., Wu C., Li J., Li X., Zhang L., He R. (2021). Susceptibility assessment for rainfall-induced landslides using a revised logistic regression method. Nat. Hazards.

[17] Khan H., Shafique M., Khan M.A., Bacha M.A., Shah S.U., Calligaris C. (2019). Landslide susceptibility assessment using Frequency Ratio, a case study of northern Pakistan. Egypt. J. Remote Sens. Space Sci..

[18] Panchal S., Shrivastava A.K. (2021). A Comparative Study of Frequency Ratio, Shannon’s Entropy and Analytic Hierarchy Process (AHP) Models for Landslide Susceptibility Assessment. ISPRS Int. J. Geo-Inf..

[19] Althuwaynee O.F., Pradhan B., Lee S. (2012). Application of an evidential belief function model in landslide susceptibility mapping. Comput. Geosci..

[20] Ilia I., Tsangaratos P. (2015). Applying weight of evidence method and sensitivity analysis to produce a landslide susceptibility map. Landslides.

[21] Chen Z., Liang S., Ke Y., Yang Z., Zhao H. (2019). Landslide susceptibility assessment using different slope units based on the evidential belief function model. Geocarto Int..

[22] Devkota K.C., Regmi A.D., Pourghasemi H.R., Yoshida K., Pradhan B., Ryu I.C., Dhital M.R., Althuwaynee O.F. (2012). Landslide susceptibility mapping using certainty factor, index of entropy and logistic regression models in GIS and their comparison at Mugling–Narayanghat road section in Nepal Himalaya. Nat. Hazards.

[23] Chen Z., Liang S., Ke Y., Yang Z., Zhao H. (2017). Landslide susceptibility assessment using evidential belief function, certainty factor and frequency ratio model at Baxie River basin, NW China. Geocarto Int..

[24] Huang Y., Zhao L. (2018). Review on landslide susceptibility mapping using support vector machines. Catena.

[25] Zhao Z., Liu Z.Y., Xu C. (2021). Slope Unit-Based Landslide Susceptibility Mapping Using Certainty Factor, Support Vector Machine, Random Forest, CF-SVM and CF-RF Models. Front. Earth Sci..

[26] Kim J.C., Lee S., Jung H.S., Lee S. (2017). Landslide susceptibility mapping using random forest and boosted tree models in Pyeong-Chang, Korea. Geocarto Int..

[27] Behnia P., Blais-Stevens A. (2017). Landslide susceptibility modelling using the quantitative random forest method along the northern portion of the Yukon Alaska Highway Corridor, Canada. Nat. Hazards.

[28] Wang S., Zhuang J., Zheng J., Fan H., Kong J., Zhan J. (2021). Application of Bayesian Hyperparameter Optimized Random Forest and XGBoost Model for Landslide Susceptibility Mapping. Front. Earth Sci..

[29] Park S.J., Lee C.W., Lee S., Lee M.J. (2018). Landslide Susceptibility Mapping and Comparison Using Decision Tree Models: A Case Study of Jumunjin Area, Korea. Remote Sens..

[30] Pham B.T., Phong T.V., Nguyen-Thoi T., Parial K.K., Singh S., Ly H.-B., Nguyen K.T., Ho L.S., Le H.V., Prakash I. (2020). Ensemble modeling of landslide susceptibility using random subspace learner and different decision tree classifiers. Geocarto Int..

[31] Aditian A., Kubota T., Shinohara Y. (2018). Comparison of GIS-based landslide susceptibility models using frequency ratio, logistic regression, and artificial neural network in a tertiary region of Ambon, Indonesia. Geomorphology.

[32] Can A., Dagdelenler G., Ercanoglu M., Sonmez H. (2017). Landslide susceptibility mapping at Ovacık-Karabük (Turkey) using different artificial neural network models: Comparison of training algorithms. Bull. Eng. Geol. Environ..

[33] Jacinth Jennifer J., Saravanan S. (2021). Artificial neural network and sensitivity analysis in the landslide susceptibility mapping of Idukki district, India. Geocarto Int..

[34] Ren T., Gong W., Gao L., Zhao F., Cheng Z. (2022). An Interpretation Approach of Ascending–Descending SAR Data for Landslide Identification. Remote Sens..

[35] Nappo N., Peduto D., Mavrouli O., van Westen C.J., Gullà G. (2019). Slow-moving landslides interacting with the road network: Analysis of damage using ancillary data, in situ surveys and multi-source monitoring data. Eng. Geol..

[36] Zhang L., Dai K., Deng J., Ge D., Liang R., Li W., Xu Q. (2021). Identifying Potential Landslides by Stacking-InSAR in Southwestern China and Its Performance Comparison with SBAS-InSAR. Remote Sens..

[37] Dong J., Zhang L., Liao M., Gong J. (2019). Improved correction of seasonal tropospheric delay in InSAR observations for landslide deformation monitoring. Remote Sens. Environ..

[38] Cai J., Liu G., Jia H., Zhang B., Wu R., Fu Y., Xiang W., Mao W., Wang X., Zhang R. (2022). A new algorithm for landslide dynamic monitoring with high temporal resolution by Kalman filter integration of multiplatform time-series InSAR processing. Int. J. Appl. Earth Obs. Geoinf..

[39] Jiang J.J., Wei W.X., Shao W.L., Liang Y.F., Qu Y.Y. (2021). Research on Large-Scale Bi-Level Particle Swarm Optimization Algorithm. IEEE Access.

[40] Moretto S., Bozzano F., Mazzanti P. (2021). The Role of Satellite InSAR for Landslide Forecasting: Limitations and Openings. Remote Sens..

[41] Zhou D., Zuo X., Zhao Z. (2022). Constructing a Large-Scale Urban Land Subsidence Prediction Method Based on Neural Network Algorithm from the Perspective of Multiple Factors. Remote Sens..

[42] Liu Z., Qiu H., Zhu Y., Liu Y., Yang D., Ma S., Zhang J., Wang Y., Wang L., Tang B. (2022). Efficient Identification and Monitoring of Landslides by Time-Series InSAR Combining Single- and Multi-Look Phases. Remote Sens..

[43] Yu S.Y., Yang Y.Y., Zhang P.F., Sun J., Luo J.J. (2020). Monitoring Land Subsidence and Fault Activity in Hefei City Based on MT-InSAR. J. Geod. Geodyn..

[44] Qu C.Y., Shan X.J., Zhang G.H., Xu X.B., Song X.G., Zhang G.F., Liu Y.H. (2014). The Research Progress in Measurement of Fault Activity by Times Series InSAR and Discussion of Related Issues. Seismol. Geol..

[45] Qiu H.J. (2012). Study on the Regional Landslide Characteristic Analysis and Hazard Assessment: A Case Study of Ningqiang County. Ph.D. Thesis.

[46] Tang G., Song X., Li F., Zhang Y., Xiong L. (2015). Slope spectrum critical area and its spatial variation in the Loess Plateau of China. J. Geogr. Sci..

[47] Zhao S., Zhao Z. (2021). A Comparative Study of Landslide Susceptibility Mapping Using SVM and PSO-SVM Models Based on Grid and Slope Units. Math. Probl. Eng..

[48] Balogun A.L., Rezaie F., Pham Q.B., Gigović L., Drobnjak S., Aina Y.A., Panahi M., Yekeen S.T., Lee S. (2021). Spatial prediction of landslide susceptibility in western Serbia using hybrid support vector regression (SVR) with GWO, BAT and COA algorithms. Geosci. Front..

[49] Hussain M.A., Chen Z., Zheng Y., Shoaib M., Shah S.U., Ali N., Afzal Z. (2022). Landslide Susceptibility Mapping Using Machine Learning Algorithm Validated by Persistent Scatterer In-SAR Technique. Sensors.

[50] Fang X., Li X., Zhang Y., Zhao Y., Qian J., Hao C., Zhou J., Wu Y. (2021). Random forest-based understanding and predicting of the impacts of anthropogenic nutrient inputs on the water quality of a tropical lagoon. Environ. Res. Lett..

[51] Belgiu M., Drăguţ L. (2016). Random forest in remote sensing: A review of applications and future directions. ISPRS J. Photogramm. Remote Sens..

[52] Rahmati O., Tahmasebipour N., Haghizadeh A., Pourghasemi H.R., Feizizadeh B. (2017). Evaluation of different machine learning models for predicting and mapping the susceptibility of gully erosion. Geomorphology.

[53] Pandey V.K., Sharma K.K., Pourghasemi H.R., Bandooni S.K. (2019). Sedimentological characteristics and application of machine learning techniques for landslide susceptibility modelling along the highway corridor Nahan to Rajgarh (Himachal Pradesh), India. Catena.

[54] Huang F., Zhang J., Zhou C., Wang Y., Huang J., Zhu L. (2020). A deep learning algorithm using a fully connected sparse autoencoder neural network for landslide susceptibility prediction. Landslides.

